# Acupuncture and related therapies for stable angina pectoris: A protocol for network meta-analysis

**DOI:** 10.1097/MD.0000000000023756

**Published:** 2020-12-18

**Authors:** Yixuan Xue, Xiaolei Zhang, Qiqi Yang, Yanji Zhang, Zhenzhen Liu, Wei Lu, Wei Huang

**Affiliations:** a College of Acupuncture-Moxibustion and Orthopaedics, Hubei University of Chinese Medicine, The Co-innovation Center for Preventive Treatment of Disease of Acupuncture-moxibustion in Hubei Province; b First Clinical College, Hubei University of Chinese Medicine, Hubei Provincial Hospital of Traditional Chinese Medicine, Wuhan, China.

**Keywords:** stable angina pectoris, acupuncture, network meta-analysis, protocol

## Abstract

**Background:**

Stable angina pectoris (SAP) is one of the important causes and harbingers of disability and mortality worldwide in the cardiovascular diseases. Acupuncture has been widely applied in the treatment and prevention of cardiovascular diseases in recent years. This systematic review protocol aims to analyze different acupuncture and related therapies to treat SAP, with a view to providing an evidence-based basis for clinical implementation of treatment for patients with SAP.

**Methods and analysis:**

The electronic databases of PubMed, EMBASE, The Cochrane Library, Chinese National Knowledge Infrastructure (CNKI), Wanfang database, Chinese Science and Technology Periodical Database (VIP), and China Biology Medicine Database (CBM) will be searched from inception to November 2020. The outcome measures were angina attack frequency, ECG changes, angina pain intensity, performance on the Six-Minute Walk Test (6-MWT) and reported adverse events. Study inclusion, data extraction and quality assessment will be performed independently by 2 reviewers. STATA 14.0 will be used to perform pairwise meta-analysis. STATA 13.0 and WinBUGS 1.4.3 will be used to perform pairwise meta-analysis and will be used to conduct network meta-analyses.

**Results:**

The results of this review will generate a comprehensive review of current evidence and be published on a peer-reviewed journal.

**Conclusions:**

The result of this network meta-analysis is expected to provide a possible ranking for acupuncture treatment methods of stable angina pectoris and offer better options for patients with stable angina pectoris.

**Ethics and dissemination:**

Ethical approval is not necessary since this protocol is only for systematic review and does not involve privacy data or conduct an animal experiment. This protocol will be disseminated by a peer-review journal or conference presentation.

**Trial registration number:**

INPLASY2020110035.

## 1 Introduction

Stable angina pectoris (SAP) is a clinical syndrome characterized by temporary and acute myocardial ischemia due to insufficient blood supply caused by organic and/or functional lesions of the coronary arteries, which leads to paroxysmal and squeezing pain in the anterior chest region.^[1]^

With the increase in age, economic development and the continuous improvement of peoples living standards, the prevalence of hypertension, hyperlipidemia, hyperglycemia, and obesity in our population is increasing, and these risk factors are increasing the morbidity and mortality of SAP. According to relevant studies in China, an average of 3.4 million people over the age of 40 are affected by SAP every year, with a prevalence rate of 9.6%, and it is closely related to the increased risk of sudden cardiac death.^[2,3]^ Without timely treatment, about 35% of patients with SAP can turn into acute myocardial infarction, and even life-threatening.^[4]^

The clinical treatment of SAP is based on the principle of reducing symptoms, improving myocardial ischemia and hypoxia, preventing dangerous events and improving quality of life. The main treatments currently available in Western medicine include drug treatment, PCI, and coronary artery grafting. Drug therapy has obvious safety limitations in the dosage and combination of drug therapy, and long-term uninterrupted oral use also causes a lot of inconvenience to patients, affecting their quality of life and standard of living.^[5]^ PCI and coronary artery grafting have high treatment costs, serious complications and have not significantly reduced the incidence of adverse cardiovascular events. Therefore, it is particularly important to find appropriate complementary and alternative treatments for patients with stable angina, such as manual acupuncture, electric acupuncture, warm needling, moxibustion, acupoint injection, acupoint application, acupoint catgut embedding, the treatment with acupuncture and medication, which are generally regarded as safe and effective treatments by the public. However, the diversity of acupuncture-related therapies and the lack of direct comparison of treatments between different acupuncture-related therapies are not conducive to clinical promotion and optimal treatment selection. Therefore, we will conduct this network meta to analyze different acupuncture and related therapies to treat SAP, with a view to providing an evidence-based basis for clinical implementation of treatment for patients with SAP.

## 2 Methods

### 2.1 Study registration

This systematic review protocol has been registered on INPLASY with number INPLASY2020110035 (https://inplasy.com/inplasy-2020-11-0035/). Additionally, the current protocol report adheres to the Cochrane Handbook for Systematic Reviews and Meta-Analysis Protocol (PRISMA-P) guidelines.^[6]^ Any change of the review will be described if needed.

### 2.2 Inclusion criteria

#### 2.2.1 Types of studies

Only randomized controlled trials (RCT) published in English and Chinese about acupuncture for stable angina pectoris will be included. Trials using a two-arm or more than two-arms parallel design will be also included.

#### 2.2.2 Types of participants

Adult men and women who had been diagnosed with stable angina pectoris according to the diagnostic criteria of ACC/AHA angina pectoris,^[7]^ regardless of their age, race or gender.

#### 2.2.3 Types of interventions

Acupuncture and related therapies including but not limited to acupuncture, electric acupuncture, warm needling, moxibustion, acupoint injection, acupoint application, acupoint catgut embedding, auricular acupuncture, regardless of time of treatment and amount of stimulation.

#### 2.2.4 Types of comparators

Control intervention will be limited to sham acupuncture or placebo, routine care, conventional drugs. When studies combine acupuncture therapies with other active therapy, both the experimental and the control groups are required to use the same active therapy.

#### 2.2.5 Types of outcome measures

##### 2.2.5.1 Primary outcomes: Angina attack frequency

##### 2.2.5.2 Secondary outcomes:

1. ECG changes (mainly ST-segment depression);
2. Angina pain intensity (assessed by visual analogue scale);
3. Performance on the Six-Minute Walk Test (6-MWT);
4. Reported adverse events.

### 2.3 Exclusion criteria

1. Case report, cross-sectional studies, comments, cohort studies, animal experiments, and reviews will be excluded.
2. People with unstable angina (pain at rest) and those with refractory angina for whom revascularisation was planned.
3. Participants who are not appropriate to receive acupuncture therapy, such as pregnant or lactating women.
4. Acupuncture therapies combined with herbs or massage will be excluded.
5. Incomplete data or information.
6. Duplicate publication.
7. No predetermined outcome index.

### 2.4 Search methods for identification of studies

#### 2.4.1 Electronic searches

We will search the following electronic databases without restrictions for language or publication status: PubMed, EMBASE, The Cochrane Library, Chinese National Knowledge Infrastructure (CNKI), Wanfang database, Chinese Science and Technology Periodical Database (VIP) and China Biology Medicine Database (CBM) from inception to November 2020. We will apply a combination of Medical Subject Heading (MeSH) and free-text terms incorporating database-specific controlled vocabularies and text words to implement search strategies. Besides, the previous relevant reviews conducted on acupuncture-related therapies for stable angina pectoris and reference lists of included studies will also be searched. And the searches will be re-run just before the final analyses to retrieve the most recent studies eligible for inclusion.

#### 2.4.2 Searching strategy

The search strategy for PubMed is shown in Table 1, which includes all search terms. Same strategy will be used in other electronic databases.

**Table 1:** Search strategy for PubMed database.

Number	Search terms
#1	angina, stable [Mesh]
#2	angina pectoris [Title/Abstract]
#3	chronic stable angina pectoris [Title/Abstract]
#4	stable angina pectoris [Title/Abstract]
#5	chronic stable angina [Title/Abstract]
#6	stable angina [Title/Abstract]
#7	angina pectoris [Title/Abstract]
#8	Angina [Title/Abstract]
#9	#1 OR #2 OR #3 OR #4 OR #5 OR #6 OR #7 OR #8
#10	Acupuncture [Mesh]
#11	Acupuncture Points [Mesh]
#12	Acupuncture Therapy [Mesh]
#13	Electroacupuncture [Mesh]
#14	Manual acupuncture [Title/Abstract]
#15	silver needle [Title/Abstract]
#16	needle pricking [Title/Abstract]
#17	warm needling [Title/Abstract]
#18	moxibustion [Title/Abstract]
#19	acupoint application [Title/Abstract]
#20	acupoint injection [Title/Abstract]
#21	Manual acupuncture [Title/Abstract]
#22	acupoint catgut embedding [Title/Abstract]
#23	auricular acupuncture [Title/Abstract]
#24	#10 OR #11 OR #12 OR #13 OR #14 OR #15 OR #16 #17 OR #18 OR #19 OR #20 OR #21 OR #22 OR #23
#25	Randomized Controlled Trial [Publication Type]
#26	controlled clinical trial [Publication Type]
#27	randomized [Title/Abstract]
#28	clinical trials as topic [mesh: noexp]
#29	Trial [Title/Abstract]
#30	#25 OR #26 OR #27 OR #28 OR #29
#31	Humans [Mesh]
#32	#30 AND #31
#33	#9 AND #24 AND #32

### 2.5 Data collection and analysis

#### 2.5.1 Selection of studies

**Figure 1:**
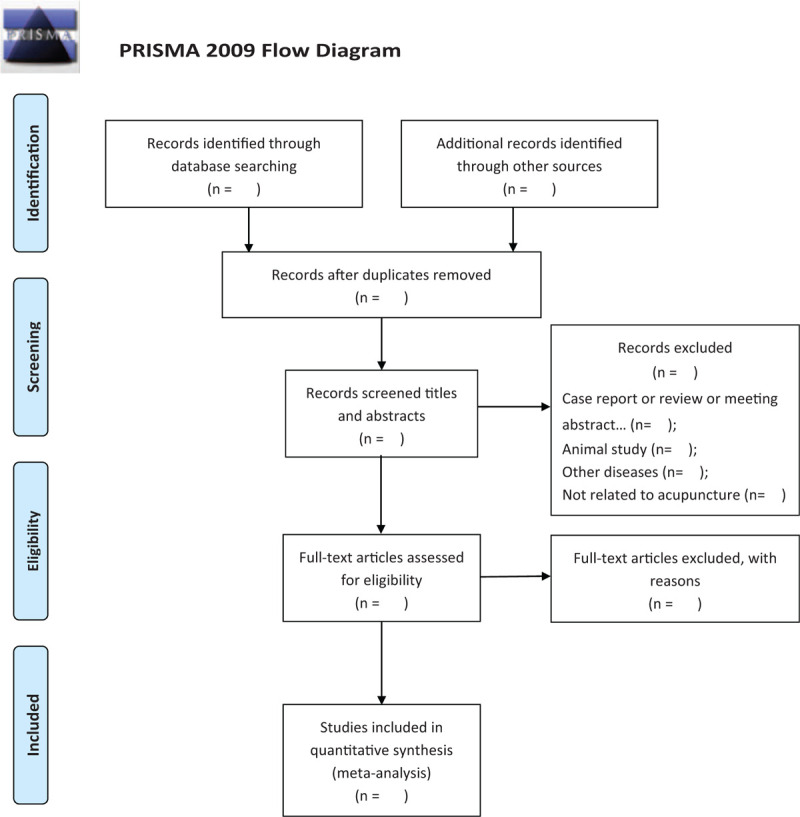
Flow diagram of the study selection process.

All reviewers will receive professional training to understand the objective and process of the review before the selection of studies. All the retrieved studies will be managed with EndnoteX7, and the duplicated studies will be discarded. Two review authors (YQQ and ZXL) screened independently titles and abstracts of studies to identify studies that potentially meet the inclusion criteria outlined above. Then the full text of these potentially eligible studies will be independently assessed for eligibility by 2 reviewers (YQQ and ZYJ). Any disagreement between them over the eligibility of studies will be discussed with the third reviewer (ZXL). The procedures of the study selection will be performed in accordance with the PRISMA flow chart (see Figure 1).

#### 2.5.2 Data extraction and management

Two independent authors (YQQ and ZXL) extracted the following details from the included studies:

1. Study characteristics (including authors, journal, publication year, method of randomization and blinding method).
2. Participants (sample size, age, duration of disease, disease diagnostic criteria, type of SAP and symptoms and signs, duration).
3. Intervention (timing and duration for treatment, acupoint selection, types and method of acupuncture procedure).
4. Outcomes (outcome measures, duration of follow-up, its intergroup differences and adverse events).

Any disagreement noticed in the process of data cross-checking will be discussed with the third reviewer. (XYX).

#### 2.5.3 Assessment of risk of bias and reporting quality of included studies

The risk of bias will be performed by 2 raters independently (YQQ and ZXL) based on the Cochrane risk of bias tool.^[8]^ It includes 7 specific domains:

1. random sequence generation,
2. allocation concealment,
3. blinding of participants and personnel,
4. blinding of outcome data,
5. incomplete outcome data,
6. selective reporting and
7. other bias.

We will manage to reach a consensus between 2 raters on condition that there is any disagreement when assessing the risk of bias of the studies. If necessary, a third rater (XYX) will resolve the disagreement.

#### 2.5.4 Dealing with missing data

If there are ambiguous or unreported data, we will contact the corresponding authors for the missing data to get specific information by email. If the complete data is still not available, then we will exclude the article of missing data from the analysis.

#### 2.5.5 Data synthesis

##### 2.5.5.1 Pairwise meta-analysis

For each direct treatment comparison, if no less than 2 RCTs, we will perform pairwise meta-analyses. We will use the odds ratio with 95% confidence intervals (CI) to assess the effect size of dichotomous variables, while the effect size of continuous variables will be assessed using the mean difference (MD). Heterogeneity between the studies in effect measures will be assessed using the *I*^2^ statistic, and we will consider an *I*^2^ value greater than 50% (*I*^2^ > 50%) as being indicative of substantial heterogeneity, and in this case, a random-effects model will be applied to process the data. Otherwise, we choose a fixed-effects model (*I*^2^ ≤ 50%). When the results are substantial heterogeneity or considerable heterogeneity, sensitivity analysis and subgroup analysis will be made to explore possible sources. If meta-analysis is impossible, data will be reported by a descriptive summary.

##### 2.5.5.2 Network meta-analyses

STATA 13.0 and WinBUGS 1.4.3 will be used to perform NMA to synthesize direct and indirect evidence. The convergence of the stimulation is tested by the potential scale reduction parameter, when the closer potential scale reduction parameter will be to 1, the better the model convergence is and more reliable the conclusion is.^[9]^ The selection of the final model will depend on the deviance information criterion value. Generally, a model with a smaller deviance information criterion value is better.^[10]^ The effect size is expressed in 95% CI, and the numerical variable is expressed as SMD. The treatment level for each result will operate on the cumulative sorting curve (SUCRA) interface. The evidence relationship incorporated into the study will be calculated by STATA. If there is a “closed loop,” the node splitting method will be used to evaluate the inconsistency of each loop.^[11]^

#### 2.5.6 Subgroup analysis

When considerable heterogeneity is detected in a previous analysis, a subgroup analysis will be performed if necessary. Subgroup analyses of the following factors will be conducted to assess heterogeneity as well as possible: age, duration of disease, type of acupuncture, the treatment of the control group.

#### 2.5.7 Publication bias

Publication bias will be evaluated using an Egger regression test which will help avoid observation bias and produce a funnel plot indicating a digitally based modeling result.

#### 2.5.8 Quality of evidence

Quality of evidence will be evaluated by the Grades of Recommendations Assessment Development and Evaluation (GRADE) guidelines. There are 3 factors (residual confounding, dose-response gradient and large magnitude of effect) to promote the quality and 5 factors (study limitations, inconsistency, indirectness, publication bias, and imprecision) to lower it and the quality will be graded in very low, low, moderate, and high. GRADE profiler 3.6 will be used to conduct the assessment.

## 3 Discussion

Previous clinical researches have shown that acupuncture therapy, in addition to standard anti-angina therapy, can reduce angina symptoms, frequency, and pain intensity in patients with stable angina pectoris.^[2,12]^ Acupuncture has been recommended as one of the treatment methods of stable angina pectoris in clinical practice.^[13]^ Therefore, it is worth promoting acupuncture in the clinical prevention and treatment of stable angina pectoris with its simplicity, safety, affectivity, and acceptability. While researchers agree there is great promise in acupuncture, it has yet to identify the optimal acupuncture treatment plan for stable angina pectoris patients.

The proposed NMA will provide evidence of the comparative effectiveness and safety of Acupuncture and related therapies for stable angina pectoris, benefiting for clinicians and guideline-makers. As we have seen, this NMA will be the first NMA in this respect. Because previous relevant reviews and meta-analyses only conduct statistical analysis of acupuncture treatment effect of stable angina pectoris and did not compare the efficacy of acupuncture and other related methods.^[14,15]^ Importantly, a recent large-scale RCT published in JAMA Intern Med have provided substantial data with respect to this topic.^[2]^ Thus, we plan to conduct this study to investigate various acupuncture methods for stable angina pectoris.

This protocol has been registered with international prospective register of systematic reviews (INPLASY2020110035). And it will follow the guidelines of Cochrane Handbook for Systematic Reviews of Interventions and the PRISMA-P statement. Moreover, the quality of evidence will be appraised by the GRADE approach. However, there are certain limitations of our NMA, including potential shortcomings of primary studies such as the presence of publication bias, high heterogeneity, and poor quality of reporting.

In a word, this study is expected to provide a possible ranking for acupuncture treatment methods of stable angina pectoris. In views of this ranking of acupuncture treatment methods, clinicians will make more accurate and optimal treatment protocols for patients with stable angina pectoris. And the findings and results of this study will be published in a peer-reviewed journal.

## Author contributions

Yixuan Xue and Xiaolei Zhang and Qiqi Yang have contributed equally to this work. All authors have read and approved the publication of the protocol.

**Conceptualization:** Yixuan Xue, Qiqi Yang, Xiaolei Zhang.

**Data curation:** Qiqi Yang, Xiaolei Zhang, Yanji Zhang.

**Formal analysis:** Wei Huang, Yixuan Xue, Qiqi Yang.

**Investigation:** Qiqi Yang, Yanji Zhang.

**Methodology:** Wei Huang, Wei Lu, Xiaolei Zhang.

**Software:** Yanji Zhang, Qiqi Yang, Zhenzhen Liu.

**Supervision:** Wei Huang, Wei Lu.

**Writing – original draft**: Yixuan Xue, Qiqi Yang, Xiaolei Zhang, Yanji Zhang.

**Writing – review & editing:** Wei Huang, Wei Lu, Xiaolei Zhang, Zhenzhen Liu.
